# Editorial: Insights in urologic oncology, volume I

**DOI:** 10.3389/fruro.2023.1240362

**Published:** 2023-08-02

**Authors:** Olivia Krivitsky, Ketan K. Badani, Trushar Patel

**Affiliations:** ^1^ Mount Sinai Department of Urology, Mount Sinai Health System, New York, NY, United States; ^2^ Department of Urology, University of South Florida, Tampa, FL, United States

**Keywords:** bladder cancer, urothelial carcinoma, prostate cancer, outcome, kidney cancer

Urologic oncology is a rapidly evolving field, and this Research Topic seeks to shed light on the latest advancements and underexplored methods that hold great potential for advancing the field and guiding future research. Various topics of significance will be examined, including the role of androgen receptor expression in determining the severity of urothelial carcinoma, the impact of nephroureterectomy on bladder tumor recurrence, the diagnostic and therapeutic potential of prostate-specific membrane antigen (PSMA) in advanced prostate cancer, and the progress made in robotic-assisted partial nephrectomy. While the effectiveness of existing practices for detecting and treating urological cancers varies, this editorial aims to explore novel methodologies that have the potential to enhance accuracy in diagnosis and treatment.

The first article, authored by Belbina et al., focuses on the diagnostic and therapeutic benefits of employing PSMA in the detection and treatment of advanced prostate cancer. Through an extensive systematic review, the study concludes that utilizing PSMA as a diagnostic tool and therapeutic marker is not only effective, but also aids in reducing the costs associated with biochemical recurrence in prostate cancer patients compared to conventional practices.

Further research is warranted to fully comprehend the role of PSMA in bridging the existing gap in advanced prostate cancer diagnosis and treatment.

Furthermore, Szabados et al. delve into the role of androgen receptor expression as a negative prognostic indicator in metastatic urothelial carcinoma (mUC), building upon previous preclinical and clinical studies that have demonstrated a robust correlation between AR signaling and bladder tumorigenesis. The samples utilized in this investigation were acquired from the LaMB phase III clinical trial (NCT00949455). This trial aimed to evaluate the effectiveness of lapatinib versus a placebo following initial platinum-based chemotherapy in individuals with HER 1/2-positive metastatic urothelial carcinoma of the bladder. The findings suggest that AR expression may hold prognostic value in metastatic urothelial carcinoma, with higher AR expression associated with poorer outcomes in terms of progression-free survival and overall survival. Further research and validation beyond the scope of the LaMB study are necessary to confirm the prognostic value of AR expression in the treatment of urothelial carcinoma. While both Belbina et al. and Szabados et al. focus on specific biomarkers in the prognosis and treatment of urologic oncology, the next two articles within this Research Topic shift the focus towards the benefits and merits in different urological surgical practices.

Miyagi et al. discuss bladder tumor recurrence after radical nephroureterectomy for upper tract urothelial carcinoma, where recurrence rates range from 22% to 47% within the first year after radical nephroureterectomy for this condition. This article primarily focuses on identifying predictors of recurrence and proposing methods to mitigate the risk. The authors categorize these risk factors as either modifiable or non-modifiable, with treating urologists having the greatest potential to decrease recurrence by addressing treatment-specific risks. To minimize the likelihood of recurrence, several approaches can be implemented. These include restricting diagnostic ureteroscopy with biopsy to cases where the diagnosis is uncertain, employing perioperative intravesical chemotherapy with additional research to determine optimal timing, and conducting thorough distal ureterectomy with bladder cuff excision.

Expanding on the surgical practices discussed by Miyagi et al., Faria et al. aimed to reach a consensus among highly experienced surgeons from referral centers in Brazil regarding the different technical complexities and principles associated with robotic-assisted partial nephrectomy (RAPN), considered the “gold standard” approach for treating small renal masses. Through an online questionnaire consisting of 131 questions, the researchers addressed topics such as “preoperative settings, surgical technique, pathological analysis, technology usage, and challenging cases.” The study produced recommendations based on consensus, which highlighted the significance of certain practices. These included the necessity of conducting initial RAPN cases with the guidance of proctors, utilizing high-quality imaging exams for preoperative planning, minimizing the extent of renal parenchyma removal, and ensuring effective hemostatic suturing techniques while minimizing renal parenchyma ischemia. These recommendations aim to improve the understanding and practice of RAPN among surgeons, taking into account the available literature and guidelines. Although both articles address different surgical procedures and topics, they are interconnected as they contribute to the understanding and enhancement of urological surgical practices.

In conclusion, the articles reviewed in this Research Topic highlight important findings and recommendations in the field of urologic oncology. These articles contribute to the advancement of urological oncology by shedding light on biomarkers, prognostic indicators, and surgical techniques, emphasizing the importance of ongoing research and collaboration among healthcare professionals. In summary, this Research Topic aims to provide valuable insights into crucial areas of urologic oncology and encourages further research to advance our understanding and improve the detection and treatment of urologic cancers.

## Author contributions

All authors listed have made a substantial, direct, and intellectual contribution to the work and approved it for publication.

